# A multifactorial interdisciplinary intervention reduces frailty in older people: randomized trial

**DOI:** 10.1186/1741-7015-11-65

**Published:** 2013-03-11

**Authors:** Ian D Cameron, Nicola Fairhall, Colleen Langron, Keri Lockwood, Noeline Monaghan, Christina Aggar, Catherine Sherrington, Stephen R Lord, Susan E Kurrle

**Affiliations:** 1 Rehabilitation Studies Unit, University of Sydney, PO Box 6, Ryde NSW 1680, Australia; 2 George Institute for Global Health, University of Sydney, PO Box M201 Missenden Road, Sydney NSW 2050, Australia; 3 Division of Rehabilitation and Aged Care, Hornsby Ku-ring-gai Health Service, Palmerston Road, Hornsby NSW 2077, Australia; 4 Faculty of Nursing and Midwifery, University of Sydney, Camperdown NSW 2006, Australia; 5 Falls and Balance Research Group, Neuroscience Research Australia, University of New South Wales, PO Box 1165, Randwick NSW 2031, Australia

**Keywords:** activities of daily living, frail elderly, randomized controlled trial, therapeutics, walking

## Abstract

**Background:**

Frailty is a well known and accepted term to clinicians working with older people. The study aim was to determine whether an intervention could reduce frailty and improve mobility.

**Methods:**

We conducted a single center, randomized, controlled trial among older people who were frail in Sydney, Australia. One group received an intervention targeting the identified characteristics of frailty, whereas the comparison group received the usual health care and support services. Outcomes were assessed by raters masked to treatment allocation at 3 and 12 months after study entry. The primary outcomes were frailty as assessed by the Cardiovascular Health Study criteria, and mobility as assessed by the Short Physical Performance Battery. Secondary outcome measures included disability, depressive symptoms and health-related quality of life.

**Results:**

A total of 216 participants (90%) completed the study. Overall, 68% of participants were women and the mean age was 83.3 years (standard deviation, 5.9). In the intention-to-treat analysis, the between-group difference in frailty was 14.7% at 12 months (95% confidence interval: 2.4%, 27.0%; *P *= 0.02). The score on the Short Physical Performance Battery, in which higher scores indicate better physical status, was stable in the intervention group and had declined in the control group; with the mean difference between groups being 1.44 (95% confidence interval, 0.80, 2.07; *P <*0.001) at 12 months. There were no major differences between the groups with respect to secondary outcomes. The few adverse events that occurred were exercise-associated musculoskeletal symptoms.

**Conclusions:**

Frailty and mobility disability can be successfully treated using an interdisciplinary multifaceted treatment program.

**Trial registration:**

Australia and New Zealand Clinical Trials Register (ANZCTR): ACTRN12608000250336

## Background

Frailty is a key theme in the aging literature, with research to date allowing an increased understanding of the definition, biological basis and associations of frailty [1]. Care of frail individuals is hindered, however, by the lack of clear consensus on how frailty should be assessed and diagnosed in the clinical setting [2,3]. Furthermore, frailty is usually comorbid with multiple medical conditions. Vulnerability to deterioration can render care of the frail person difficult, given the generally fragmented and underfunded nature of current health delivery models, particularly in the community setting.

Two main definitions of frailty are currently accepted. The Cardiovascular Health Study (CHS) Frailty Phenotype [1] diagnoses people as frail if they meet pre-determined values for three or more of five criteria: slow gait speed, weak grip strength, exhaustion, low energy expenditure, and weight loss. Alternatively, the use of a Frailty Index can measure the deficits present in an individual as a proportion of all potential deficits across multiple domains (for example, chronic diseases, mood, social resources, cognition) [4]. Other measures of frailty include using a combination of instruments, each measuring a single aspect of frailty [5].

An increasing number of studies have defined the syndrome of frailty and measured its prevalence. Though several intervention studies have also been conducted in which frail older people have participated, few have recruited participants based on a specific definition of frailty [6]. Furthermore, to our knowledge, no interventions have been developed to specifically reverse the syndrome of frailty and there are no intervention studies that have had frailty (specifically defined) as a primary outcome. We propose that, by conducting a multifactorial intervention targeting each component of frailty shown to be amenable to modification in previous studies in frail older people, frailty can be reduced.

We sought to compare the effects of a multifactorial, interdisciplinary intervention specifically targeting frailty with usual care. The study aimed to establish the effects of the intervention on both frailty and impaired mobility. The hypotheses were that the multifactorial, interdisciplinary intervention would reduce frailty assessed with a frailty phenotype score [1], improve mobility as measured with the Short Physical Performance Battery (SPPB) [7], and have a positive effect on a number of secondary outcome measures including disability, depressive symptoms and health-related quality of life.

## Methods

### Study design

We conducted a single center, randomized, controlled trial among older people who were frail in Sydney, Australia. The trial protocol has been previously reported [8]. The Northern Sydney & Central Coast Health Human Research Ethics Committee has approved the study protocol - Research Protocol Number 0709-191M - and the trial is registered with the Australian New Zealand Clinical Trials Registry: ACTRN12608000250336. Trial registration was delayed and 8% of the participants had been randomized at the time of registration. Participants provided written informed consent before randomization.

### Study population, screening and randomization

Eligible participants were identified from older people treated by clinicians working within the Division of Rehabilitation and Aged Care Services (DRACS) at Hornsby Ku-ring-gai Health Service (Sydney, Australia). DRACS is a large clinical service that has programs operating both in the community and hospital settings. Eligible participants first completed their usual treatment program before being approached to enter the study. Following participation agreement, informed consent was sought, often in conjunction with relatives. If granted, the study nurse screened for inclusion criteria. These were: adults aged 70 years or older with three or more of the CHS frailty criteria [1]; not usually living in a residential aged care facility; residing in the Hornsby or Ku-ring-gai local government areas; without moderate or severe cognitive impairment (defined as a Mini Mental State Examination score of ≤18) [9]; not an ongoing client of DRACS; without an illness likely to be associated with a life expectancy of <12 months, estimated by a score of ≤3 on a modified version of the Implicit Illness Severity Scale [10]; and not participating in another physical intervention research project. All randomized participants met the eligibility criteria.

### Interventions

Participants in the intervention group received a multifactorial, interdisciplinary treatment program intended to target frailty for a 12-month period following randomization. The interventions were individually tailored to each participant based on their frailty characteristics as assessed at baseline, and additional problems as identified during a detailed assessment by the two experienced physiotherapists providing the intervention program. Geriatric evaluation and management principles underpinned both the assessment and intervention [11].

Details of the approach to intervention are described in the protocol paper [8]. To summarize, the CHS frailty components that were present in each participant were specifically targeted [12]. If the participant met the weight loss criterion, a dietician evaluated nutritional intake. Home-delivered meals were recommended if appropriate clinical criteria applied. In addition, if the participant's body mass index was <18.5 kg/m^2^, or mid-upper arm circumference was <the 10th percentile (using Australian age and gender specific norms), nutritional supplementation was offered using commercially available, high energy, high protein supplements.

If the exhaustion criterion was met and the Geriatric Depression Scale [13] score was high, the study team considered referral to a psychiatrist or psychologist. Where the participant was socially isolated, options to encourage greater social engagement were identified, such as participation in day activity groups and telephone contact with a volunteer.

Participants who met the weakness, slowness or low energy expenditure criteria received up to 10 home-based physiotherapy sessions and performed a home exercise program, over the course of 12 months. The Weight-bearing for Better Balance (WEBB) program [14], designed to improve mobility, increase physical activity and prevent falls, was tailored to individual's physical impairments, prescribed three to five times per week, and reviewed regularly. Two physiotherapy sessions targeted the participant's mobility goal. Equipment was also recommended as necessary.

Case management by the physiotherapist, and regular case conferences involving the physiotherapist, geriatrician, rehabilitation physician, nurse and dietician, facilitated coordination of the delivery of the intervention. Reassessment was ongoing throughout the intervention phase. The physiotherapist was the co-ordinator of the intervention. Home visits usually involved several intervention components and included not only the WEBB exercise program, but other identified interventions that were relevant to the frail person at that particular time.

For all participants, additional interventions were then provided or recommended based on a comprehensive geriatric evaluation, for example review by the study geriatrician or rehabilitation physician, follow-up of chronic diseases, treatment of pain, and management of other identified conditions such as urinary incontinence.

The major limiting factor in implementing the intervention as planned was the limited ability of the participants to be able to adhere with the recommended intervention plan. The physiotherapists coordinating the intervention were careful not to overwhelm the participants with complex treatment plans and, in addition, participants often declined specific interventions.

There was a standardized approach to interventions in the study based on the study protocol and regular case discussions of each participant. The physiotherapist primarily responsible for each participant documented adherence to the study protocol and estimated a global level of adherence (in five categories) over the 12-month intervention period.

Usual care, as received by the control group in this study, consisted of those health and aged care services that would normally be available to older people. These include general practitioner and medical specialist consultations, and nursing and allied health interventions as appropriate. Australia has a system of universal health insurance so that all of its population has access to health care without significant cost. Aged care services include assistance with housekeeping and personal care and these types of services are also heavily subsidized by the Australian government for older people with care needs. Northern Sydney has a well-developed system of health and aged care that has been operating in its current form for more than 20 years.

### Outcomes

The primary outcomes measured were frailty and mobility. Frailty was assessed using the CHS definition of the frailty syndrome [1]. See Additional file 1 for details of frailty assessment criteria. Mobility was measured using the SPPB [7], which assesses the ability to stand (for 10 s) with the feet together in the side-by-side, semi-tandem and tandem positions; time to walk 4 meters; and time to rise from a chair and return to the seated position five times. The SPPB score and the lower extremity continuous summary performance score were calculated [15].

Secondary outcomes included hospitalizations and admissions to nursing care facilities that were reported on monthly calendars and confirmed from either hospital records or the relevant facility; disability was measured with the Barthel Index [16], and health-related quality of life measured by the EuroQol-5D (EQ-5D) [17]. Psychological status was assessed using the Geriatric Depression Scale (short form) [13] and deaths were recorded and verified by hospital records.

We initially chose the Timed Up and Go measure as a co-primary outcome and included this on the Clinical Trials Registry. However, early in the study we recognized that it was not feasible to collect data for this outcome and hence determined that the SPPB and Frailty would be the co-primary outcome measures. These are the co-primary outcomes recorded in the study protocol paper [8].

The data were collected in the participants' homes by experienced research nurses trained specifically for the trial. Training in the assessment tools was provided, and joint sessions conducted to standardize the administration of the assessment tools, ensuring consistent interpretation of the data recorded. Inter-rater reliability checks were conducted at commencement and mid-way through the trial.

The study aimed to recruit approximately 230 participants, to detect a clinically and statistically significant 15% difference in mobility as assessed by the lower extremity continuous summary performance score between the two groups (power = 80%, *P *= 0.05, dropouts = 15%, non-compliance = 15%, standard deviation (SD) = 0.7).

A permuted block randomization approach was used to achieve balanced treatment allocation. There were two strata (frail with three CHS frailty criteria and very frail with four or five CHS frailty criteria). A random number sequence was generated for the order of treatment allocation within the blocks using SPSS v15 RV.UNIFORM function (SPSS, Inc., Chicago, IL, USA). Block sizes of four and six were used and these blocks were randomly arranged within blocks of ten.

Project personnel not involved in assessing participants or in providing the intervention managed the randomized group allocation. The treatment allocation tables for both strata were stored centrally off site.

Staff members performing the outcome assessment and data analysis were masked to group allocation. It was not possible to blind participants and staff administering the interventions to group allocation. The number of participants inadvertently unblinding the outcome assessors was recorded.

### Harms

Adverse events were monitored and recorded by the treating clinicians. Deaths were monitored by the study statistician. A data monitoring procedure was implemented but, after consultation with the independent epidemiologist monitoring the study, interim analyses were not performed because of the limited number of adverse events.

### Statistical analysis

The study design assessed study participants at baseline, at 3 months, and at 12 months after randomization. Data were coded to permit blinding to group allocation in the statistical analysis. The primary analyses were undertaken in accordance with the intention-to-treat principle. Frailty was treated as both a dichotomous (that is, more than three CHS criteria met or not) and continuous variable, and other study outcomes as continuous variables. The chi-square test was used for frailty as a dichotomous variable, and linear regression models with baseline values as a covariate were used for continuous outcomes. We report between-group differences in percentages, or mean, with 95% confidence intervals (CIs) at the 3- and 12-month follow-ups. We tested whether the pattern of changes in frailty and mobility were modified by frailty severity at baseline, by including an interaction term of study groups with frailty severity at baseline in the regression analyses. Secondary analyses were also carried out to explore the effect of different rates of adherence (as a category variable: <25%, 25% to 49%, 50% to 74% and ≥75%) on the outcomes in the intervention group at the 12-month follow-up. The Cox regression model was used to assess time to admission for those permanently admitted to aged care facilities.

## Results

### Characteristics of the study population

**Figure 1:**
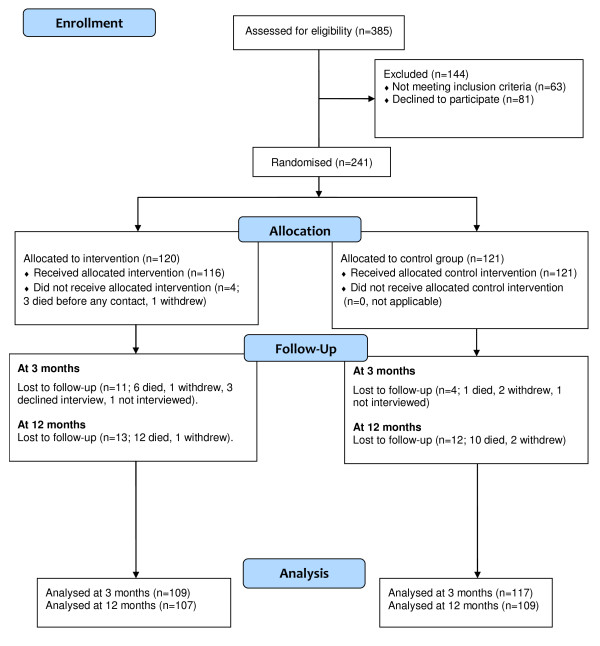
**Eligibility, randomization and follow-up**.

Participant flow is shown in Figure 1. Of the eligible people approached, 75% (241 out of 322) agreed to participate in the trial. Recruitment commenced in January 2008 and concluded in April 2010. Follow-up concluded in June 2011.

Participant characteristics are shown in Table 1. Women constituted 68% of participants, and the mean age was 83.3 years. The groups were well matched at baseline except that the control group had a slightly better SPPB mean score.

**Table 1:** Baseline characteristics of the study population

	Intervention group (n = 120)	Control group (n = 121)	*P*^a^
**Characteristic **			
Male (%)	39 (33%)	39 (32%)	0.96
Age (year)	83.4 ±5.81	83.2 ±5.91	0.80
Lives alone (%)	60 (50%)	51 (42%)	0.22
Number of frailty criteria present^b ^(%)			0.84
Three	77 (64%)	79 (65%)	
Four	33 (28%)	30 (25%)	
Five	10 (8%)	12 (10%)	
Weight	68.5 (15.7)	69.3 (17.3)	0.70
Exhaustion (%)	77 (64%)	76 (63%)	0.83
Grip strength (kg)	15.8 (7.2)	15.3 (7.2)	0.70
Gait speed (m/s)	0.48 (0.18)	0.50 (0.17)	0.32
Low physical activity (%)	73 (61%)	83 (69%)	0.21
Frailty, mean Cardiovascular Health Study score **Health status**	3.44 (0.65)	3.45 (0.67)	0.96
Coexisting conditions^c^	5.87 ±2.33	5.75 ±2.24	0.70
Body mass index (kg/m^2^)	26.1 ±5.91	26.4 ±6.04	0.67
Geriatric Depression Scale^d,e^	4.76 ±3.18	5.06 ±3.19	0.47
Mini Mental State Examination^d,e^	26.6 ±2.59	25.9 ±3.14	0.07
**Functioning**			
Disability, Barthel Index^e^	93.9 ±11.1	92.5 ±14.3	0.40
Mobility, Short Physical Performance Battery^e^	5.21 ±1.89	5.74 ±2.12	0.04
Mobility, lower extremity continuous summary performance score^e^	1.74 ±0.43	1.86 ±0.45	0.05
Health-related quality of life, EuroQol-5D^e^	7.67 ±1.47	7.83 ±1.50	0.39
Health-related quality of life, EuroQol-5D VAS^e^	58.2 ±15.8	57.9 ±18.4	0.90

Data presented as number (%) or mean ±standard deviation. ^a^*P*-values were derived from the chi-square test or analysis of variance. ^b^Frailty phenotype as specified using Cardiovascular Health Study criteria. ^c^Self-reported, doctor-diagnosed medical conditions. ^d^Missing data for Geriatric Depression Scale (n = 1), Mini Mental Status Examination (n = 2). ^e^The Geriatric Depression Scale (short form) has scores between 0 and 15 with a higher score indicating more depressive symptoms. For the Mini Mental Status Examination, higher scores indicate higher cognitive function with a maximum score of 30. The Barthel Index has scores between 0 and 100 with higher scores indicating better basic activities of daily living functioning. The Short Physical Performance Battery [7] has scores between 0 and 12, and the lower extremity continuous summary performance [15] scale has scores between 0 and 2.71; in both, better mobility function is indicated by a higher score. The EQ-5D has scores between 5 and 15 with higher scores indicating worse health-related quality of life. The EQ-5D VAS has scores between 0 and 100 with higher scores indicating better health-related quality of life. VAS: visual analog scale.

At 3 months, follow-up data from 109 out of 120 of the intervention group and 117 out of 121 of the control group were available for analysis (Figure 1). At 12 months, the availability of these follow-up data was 107 out of 120 and 109 out of 121 respectively. The follow-up attrition rate was 10.4%, with 22 of the 25 losses due to death.

Interventions:The intervention and control treatments were implemented as planned. Adherence was 0% for 16 participants (13%), including three people who died before the intervention could commence. The median adherence overall was in the category of 26% to 50%.

There was a median of 10 face-to-face sessions with a physiotherapist for each participant in the intervention group, including a median of eight sessions to teach the WEBB program. In addition, there was a median of four telephone calls to each participant and a median of four telephone calls to other parties. The WEBB program was delivered to 93% of intervention group participants. A dietetic assessment and intervention was provided to 50% of participants (resulting in 29% being recommended nutritional supplements). A medical specialist consultation (with a geriatrician or rehabilitation physician) was arranged for 24% of participants. Referral to a psychologist or psychiatrist was arranged for 3% of participants. Further details of the intervention as implemented are available from the authors on request.

### Primary outcomes

There was a lower prevalence of frailty in the intervention group compared with the control group at 12 months (absolute difference 14.7%; 95% CI: 2.4%, 27.0%; *P *= 0.02; number needed to treat = 6.8). Expressing frailty as a number of the five frailty criteria showed similar results (Table 2). Between-group differences in frailty were statistically significant at 12 months but not at 3 months. At 12 months, the average reduction in the number of frailty criteria was 0.80 (SD = 1.19) in the intervention group and 0.41 (SD = 1.02) in the control group (between-group difference 0.41; 95% CI, 0.14, 0.68; *P *<0.01).

**Table 2:** Effects of the intervention on primary and secondary outcomes, intention-to-treat analyses

	Intervention group	Control group	Percentage difference between groups or coefficient^a ^	*P*^b ^
**Primary outcomes**				
***Frailty***^c^				
3 months (number (%))	69 out of 108 (64%)	88 out of 117 (75%)	-11.3% (-23.3% to 0.7%)	0.07
12 months (number (%))	66 out of 107 (62%)	81 out of 106 (76%)	-14.7% (-27.0% to -2.4%)	0.02
Change from 0 to 3 months	108, 0.56 ±1.10	117, 0.39 ±0.92	-0.18 (-0.43 to 0.08)	0.17
Change from 0 to 12 months	107, 0.80 ±1.19	106, 0.41 ±1.02	-0.41 (-0.68 to -0.14)	<0.01
***Mobility, Short Physical Performance Battery***^c^				
3-month mean 12-month mean Change from 0 to 3 months Change from 0 to 12 months	107, 5.40 ±2.32 107, 5.83 ±2.82 107, -0.15 ±1.89 107, -0.52 ±2.47	116, 5.72 ±2.30 108, 4.69 ±2.91 116, 0.01 ±1.72 108, 0.98 ±2.30	0.05 (-0.42 to 0.51) 1.44 (0.80 to 2.07)	0.85 <0.001
***Mobility, lower extremity continuous summary performance score***^c^				
3-month mean 12-month mean Change from 0 to 3 months Change from 0 to 12 months	107, 1.72 ±0.57 107, 1.77 ±0.59 107, 0.04 ±0.49 107, 0.00 ±0.50	116, 1.80 ±0.52 108, 1.49 ±0.75 116, 0.06 ±0.38 108, 0.35 ±0.63	-0.00 (-0.12 to 0.11) 0.34 (0.19 to 0.49)	0.96 <0.001
**Secondary outcomes**				
***Barthel Index***^c^				
3-month mean 12-month mean Change from 0 to 3 months Changes from 0 to 12 months	108, 94.2 ±11.2 106, 89.5 ±17.5 108, 0.56 ±7.92 106, 5.56 ±14.61	117, 93.2 ±13.9 108, 86.1 ±24.7 116, -0.80 ±10.87 107, 6.14 ±20.76	-0.68 (-3.05 to 1.68) 0.67 (-4.23 to 5.56)	0.57 0.79
***Geriatric Depression Scale***^c^				
3-month mean 12-month mean Change from 0 to 3 months Change from 0 to 12 months	108, 4.89 ±3.14 106, 4.62 ±3.33 108, -0.19 ±2.30 106, 0.11 ±2.27	117, 4.90 ±3.24 108, 4.98 ±3.16 117, 0.13 ±2.52 108, 0.02 ±2.90	0.22 (-0.37 to 0.82) -0.18 (-0.83 to 0.47)	0.46 0.59
***EQ5D VAS***^c^				
3-month mean 12-month mean Change from 0 to 3 months Change from 0 to 12 months	108, 60.6 ±20.1 107, 57.5 ±20.8 108, -1.81 ±15.65 107, 0.41 ±18.93	117, 60.3 ±16.9 108, 57.7 ±19.7 117, -1.96 ±16.77 108, 1.12 ±20.62	0.04 (-3.93 to 4.00) 0.30 (-4.59 to 5.18)	0.99 0.91

Data presented as number, mean, ±standard deviation, unless otherwise stated. ^a^Coefficient from a linear regression model with follow-up values as a dependent variable and baseline values as a covariate. ^b^*P *values, which were derived from chi-square test or linear regression models with baseline values as a covariate, are for the differences in rate or mean between intervention and control group. ^c^The Geriatric Depression Scale (short form) has scores between 0 and 15 with a higher score indicating more depressive symptoms. For the Mini Mental Status Examination, higher scores indicate higher cognitive function with a maximum score of 30. The Barthel Index has scores between 0 and 100 with higher scores indicating better basic activities of daily living functioning. The Short Physical Performance Battery [7] has scores between 0 and 12, and the lower extremity continuous summary performance [15] scale has scores between 0 and 2.71; in both, better mobility function is indicated by a higher score. The EQ-5D (health-related quality of life) has scores between 5 and 15 with higher scores indicating worse health-related quality of life. The EQ-5D VAS has scores between 0 and 100 with higher scores indicating better health-related quality of life. VAS: visual analog scale.

Mobility remained relatively stable in the intervention group, whereas it declined substantially in the control group. At 12 months, there was an average decline on the 12-point SPPB scale of 0.98 (SD = 2.30) in the control group and an average increase of 0.52 points (SD = 2.47) in the intervention group (between-group difference 1.44 points; 95% CI: 0.80, 2.07; *P *<0.001). A similar result was seen in mobility when assessed using the lower extremity continuous summary performance score (Table 2).

### Secondary outcomes

There were no major differences between the groups with respect to the secondary outcomes of the trial (see Table 2). The number of deaths in the intervention group was 12 (10.0%), with 10 in the control group (8.26%) (*P *= 0.64). A large number of hospital admissions occurred, (intervention 74, control 67) with no significant differences between the groups (*P *= 0.32). There were similar numbers of permanent admissions to nursing care facilities in both groups (intervention 16, control 21) and, compared with the control group, the hazard ratio of time to admission for the intervention group was 0.69 (95% CI: 0.35, 1.33; *P *= 0.27).

Changes in the individual components of the frailty phenotype and the SPPB are summarized in Tables 3 and 4. We performed analyses based on adherence to the intervention (see Table 5). Table 5 shows that higher adherence was strongly associated with improved outcomes, in particular the primary outcomes.

**Table 3:** Effects of the intervention on components of frailty by intention-to-treat analyses:

	Number	Intervention group (n = 120)	Control group (n = 121)	Percentage difference between groups or coefficient^a ^	*P*^b ^
**Frailty criteria**					
***Weight (kg)***					
Change from 0 to 3 months	224	0.40 ±3.72	0.92 ±3.59	0.51 (-0.45 to 1.48)	0.29
Change from 0 to 12 months	210	0.72 ±5.98	1.48 ±5.09	0.78 (-0.72 to 2.28)	0.31
***Exhaustion ***					
Rate at 3 months (number (%))	225	47 (44%)	46 (39%)	4.2% (-8.7% to 17.1%)	0.52
Rate at 12 months (number (%))	213	29 (27%)	35 (33%)	-5.9% (-18.2% to 6.4%)	0.35
***Grip strength (kg)***					
Change from 0 to 3 months	225	0.78 ±3.56	1.02 ±4.93	0.50 (-0.55 to 1.54)	0.35
Change from 0 to 12 months	213	0.93 ±4.62	1.88 ±5.75	1.18 (-0.13 to 2.49)	0.08
***Gait speed (meter/second)***					
Change from 0 to 3 months	225	-0.006 ±0.155	-0.007 ±0.169	-0.004 (-0.046 to 0.039)	0.87
Change from 0 to 12 months	213	-0.049 ±0.183	0.019 ±0.230	0.068 (0.012 to 0.123)	0.02
***Low physical activity***					
*Rate at 3 months - No. (%)*	225	59 (55%)	74 (63%)	-8.6% (-21.4% to 4.2%)	0.19
*Rate at 12 months - No. (%)*	213	67 (63%)	80 (76%)	-12.9% (-25.2% to -0.6%)	0.04

Data presented as mean, ±standard deviation, unless otherwise stated. ^a^Coefficient from a linear regression model with follow-up values as a dependent variable and baseline values as a covariate. ^b^*P *values, which were derived from linear regression models with baseline values as a covariate, are for the differences in mean between intervention and control group. Percentages are calculated based on number of participants available for follow-up.

**Table 4:** Effects of the intervention on components of the Short Physical Performance Battery by intention-to-treat analyses

	Number	Intervention group (n = 120)	Control group (n = 121)	Coefficient^a ^	*P*^b ^value
**Short Physical Performance Battery criteria**					
***Balance score***					
Change from 0 to 3 months	223	0.19 ±1.13	0.21 ± 1.02	-0.06 (-0.32 to 0.20)	0.63
Change from 0 to 12 months	215	-0.11 ±1.24	0.59 ±1.25	0.63 (0.32 to 0.95)	<0.001
***Measured walks score ***					
Change from 0 to 3 months	223	-0.07 ±0.80	-0.08 ±0.80	-0.02 (-0.22 to 0.18)	0.82
Change from 0 to 12 months	215	-0.27 ±0.90	0.08 ±0.96	0.35 (0.10 to 0.59)	0.006
***Chair stands score***					
Change from 0 to 3 months	223	-0.26 ±0.94	-0.12 ±0.87	0.04 (-0.18 to 0.27)	0.72
Change from 0 to 12 months	215	-0.14 ±1.22	0.31 ±0.98	0.33 (0.07 to 0.59)	0.01

Data given as mean, ±standard deviation. ^a^Coefficient from a linear regression model with follow-up values as a dependent variable and baseline values as a covariate.^b^*P *values, which were derived from linear regression models with baseline values as a covariate, are for the differences in mean between intervention and control group.

**Table 5:** Primary and secondary outcomes by level of adherence to intervention at 12 months

	Adherence				
Outcome^a^	<25% (n = 42)	25% to 49% (n = 17)	50% to 74% (n = 23)	75% to 100% (n = 24)	*P *
Frailty	3.24 (2.95 to 3.53)	2.23 (1.76 to 2.70)	2.28 (1.88 to 2.68)	2.12 (1.74 to 2.51)	<0.001
Short Physical Performance Battery	4.83 (4.14 to 5.53)	5.58 (4.47 to 6.69)	6.14 (5.21 to 7.08)	7.43 (6.53 to 8.34)	<0.001
Lower extremity continuous summary performance score	1.59 (1.44 to 1.73)	1.74 (1.50 to 1.97)	1.91 (1.71 to 2.11)	1.96 (1.77 to 2.15)	0.01
Barthel Index	86.3 (81.8 to 90.8)	91.0 (83.8 to 98.2)	94.0 (87.9 to 100)	91.4 (85.5 to 97.4)	0.21
Geriatric Depression Scale	5.44 (4.78 to 6.11)	4.16 (3.09 to 5.23)	4.32 (3.42 to 5.22)	3.95 (3.07 to 4.83)	0.03
Health-related quality of life (EuroQol-5D visual analog scale)	52.2 (46.9 to 57.6)	51.2 (42.6 to 59.8)	60.0 (52.8 to 67.2)	69.4 (62.3 to 76.4)	0.001

Data presented as adjusted means (95% confidence intervals), adjusted for age, sex, living alone, number of coexisting conditions, Mini Mental State Examination score and baseline values. ^a^Variables as described in the footnote to Table 1.

A number of participants (169 out of 241, 70%) inadvertently disclosed their treatment status to the assessors. At the 12-month follow-up, disclosure of treatment status occurred in 51% of cases. We analyzed SPPB scores by whether the rater was unblinded or not, and there was no significant difference in the scores (data not shown).

### Harms

No major adverse events specifically attributable to the intervention were evident. Two participants experienced back pain that met the adverse event criterion reported in a previous study [18] and required modification of their exercise program.

## Discussion

The intervention reduced frailty and improved mobility in older people who met the CHS frailty criteria. The improvement in these primary outcomes contrasts with the non-statistically significant changes in the secondary outcomes. The lack of changes in secondary outcomes may relate to limited power to detect changes using these measures (see Table 5). However, the intervention resulted in a reduction in mobility-related disability [19].

The benefit of the intervention was not evident at 3-month follow-up and became apparent only at 12 months. This indicates that an intervention treating frailty needs to be prolonged. The analyses show participants who had higher levels of adherence to the intervention had much greater effects after adjusting for possible confounders. We acknowledge that such analyses need to be interpreted with caution [20].

In the months before participating in the study, 73% of the participants had been hospitalized. A likely explanation for the initial improvement in frailty in both the intervention and control groups (25% of control participants became non-frail by the 3-month follow-up) is that these participants were still recovering from illness. After 3 months, the frailty and mobility status of the intervention group was relatively stable whereas that of the control group had deteriorated.

### Strengths and limitations of study

The trial was completed in accordance with the published protocol. It was a pragmatic randomized trial that included participants meeting a widely accepted definition of frailty and had few exclusion criteria. In keeping with studies of this type, it was not possible to blind participants and treating clinicians to the intervention. Outcome assessors were blinded but many participants inadvertently disclosed their treatment status. The components of the frailty definition are partly self-reported and partly performance based, but the co-primary outcome (the SPPB) is a performance-based measure that should reduce observer bias.

The lack of a control group providing a sham intervention could be seen as a limitation and our trial does not tell us whether the nature of the contact with the staff providing the intervention was important. It is unlikely that such contacts could have specific effects on frailty and mobility, but could have had an effect on mood that may influence the exhaustion criterion in the CHS frailty phenotype [21]. However, we consider our trial to be comparative effectiveness research in which we are comparing the two options of usual care versus potential care through this program.

Adverse events were minor and responded to a change in the prescribed exercise intervention. Skilled physiotherapists tailored and delivered the intervention. Dropouts were not due to adverse events but were rather related to participants' beliefs or to major changes in their health conditions.

Generalizability:The study was conducted in a relatively affluent country with well-developed health and care services for older people. It used a specific clinical team with considerable experience and knowledge of the locally available aged care services. It should be feasible to generalize the intervention to other situations with similar health and care services. It is a moderate intensity intervention that could be provided as a program through an aged care health service. The intervention costs about a quarter of the amount of the program currently administered by the Australian government, designed to assist a similar group of older people in the transition from hospital to home or nursing care facility [22].

### Comparison with other studies

Trials involving older people with reduced functioning, labeled as 'frail', have shown variable improvements in disability and its components. A recent systematic review of exercise interventions in frail populations concluded that multicomponent exercise treatments for frail people are likely to be effective if undertaken on a regular basis over a prolonged period [6]. The geriatric evaluation and management literature has shown mixed effects with respect to overall benefit. This study adds to previous research by demonstrating that it is possible to identify frail older people for inclusion in a randomized trial, and that intervention can reduce the degree of frailty and improve mobility outcomes in this population.

In the group studied, it is difficult to disentangle 'frailty' from 'disability' because these two states coexisted in almost all participants. Although improvement in frailty was seen there was also improvement in mobility disability. The changes in frailty and mobility are similar in magnitude and represent medium effect sizes. In this study, changes in frailty and mobility appear closely linked. The measure of activities of daily living disability that was used, the Barthel Index, was close to its maximum and ceiling effects may have limited the ability to detect changes in disability as defined by this measure.

## Conclusions

This study has shown that treating frailty in older people is a realistic therapeutic goal. Ideally a multicenter study with a larger sample size should be conducted to confirm and extend the findings of this study. Future studies should also consider follow-up beyond the end of the intervention period.

Frailty and mobility disability can be successfully treated using an interdisciplinary multifaceted treatment program.

## Abbreviations

CHS: Cardiovascular Health Study; CI: confidence interval; DRACS: Division of Rehabilitation and Aged Care Services; EQ-5D: Euroqol; SD: standard deviation; SPPB: Short Physical Performance Battery; WEBB: Weight-bearing Exercise for Better Balance.

## Competing interests

The authors declare that they have no competing interests.

## Authors' contributions

IC, CS, SL and SK were the chief investigators on the Frailty Intervention Trial. All contributed to the study design, implementation and interpretation of the data. SK and IC also conceived the Frailty Intervention Trial and contributed to the delivery of the intervention. NF, CL, KL, NM and CA contributed to study design, implementation, delivery of the intervention, analysis and interpretation of the data. All authors contributed to revisions and read and approved the final manuscript.

## Pre-publication history

The pre-publication history for this paper can be accessed here:

http://www.biomedcentral.com/1741-7015/11/65/prepub

## Supplementary Material

Additional file 1 **Frailty outcome measure**. Definition of the frailty components, adapted from Cardiovascular Health Study Criteria [1] Click here for file
