# Supplementary material for: A multifactorial interdisciplinary intervention reduces frailty in older people: randomized trial
Source: BMC Med. 2013 Mar 11;11:65. doi: 10.1186/1741-7015-11-65 (PMC3751685; doi:10.1186/1741-7015-11-65)
Supplement: Additional file 1 — Frailty outcome measure. Definition of the frailty components, adapted from Cardiovascular Health Study Criteria [1] [file 1741-7015-11-65-S1.DOCX]

Additional File 1: Definition of Frailty Components in the Frailty Intervention Trial (FIT)

| Characteristic | Criteria |
| --- | --- |
| Weight loss/ Shrinking | Self-report of ≥ 4.5 kg lost unintentionally in previous 12 months or loss of ≥5% of weight in prior year by direct measurement of weight |
| Weakness | Lowest 20% in grip strength, measured using a dynamometer (Saehen Dynamometer, model SH5001). Best of three attempts used. Males scoring 30kg or less, female scoring 18kg or less meet the criteria |
| Exhaustion | Answering “a moderate amount” or “most of the time” to either of the 2 questions from the Centre for Epidemiological Studies-Depression Scale (CES-D) indicated exhaustion: “How often did you feel that everything you did was an effort in the last week?” or “How often did you feel that you could not get going in the last week?”. |
| Slowness | Time to walk four metres, with or without a walking aid, equals six seconds or more. |
| Low activity | In the past three months, weight bearing physical activity was not performed, more than four hours per day were spent sitting, and went for a short walk once per month or less. |
